# Nanoscale heat generation in a single Si nanowire

**DOI:** 10.1515/nanoph-2024-0604

**Published:** 2025-01-30

**Authors:** Jungkil Kim, Hoo-Cheol Lee, Hong-Gyu Park

**Affiliations:** Department of Physics, 34926 Jeju National University, Jeju, Republic of Korea; Department of Physics and Astronomy, and Institute of Applied Physics, Seoul National University, Seoul, Republic of Korea

**Keywords:** nanowire, heater, nanoscale, porous

## Abstract

We develop a nanoheater utilizing a single Si nanowire with a porous segment that produces localized heat. The 19-fold higher resistivity of the porous segment compared to the solid segment in the nanowire facilitates the substantial confinement of heat to the porous segment by Joule heating. The heat profiles of the nanowire are examined using scanning thermal microscopy, a direct thermal imaging technique. The profiles recorded along the longitudinal and cross-sectional axes of the nanowire reveal that heat is concentrated in the sub-micrometer region of the porous segment, whereas it is uniformly distributed along the whole axis of the homogeneous solid Si nanowire. Moreover, the HfO_2_-passivated nanowire device exhibits a temperature increase above 10 °C within a 0.4 × 1 μm^2^ area, which is advantageous compared to the 3.3 °C increase observed in the hBN-passivated device. These point heaters demonstrate considerable potential for future applications in biomedical engineering and optoelectronics.

## 1 Introduction

The rapid advancement in nanotechnology has facilitated the development of nanoscale devices that generate and detect electrical, optical, and thermal signals with enhanced sensitivity and spatial resolution [1], [2], [3], [4], [5], [6], [7], [8], [9], [10], [11]. Specifically, in thermal management devices, localized heating was achieved in regions smaller than micrometers [10], [11], [12], [13], [14], [15], [16]. The capability to activate point heaters is crucial for various applications requiring high precision, such as targeted drug delivery, biomedical therapy, and light-emitting devices [17], [18], [19], [20], [21], [22], [23].

Numerous nanoheater designs have emerged in previous work, highlighting diverse materials and configurations [10], [11], [12], [13], [14], [15], [16], [24], [25], [26], [27], [28], [29]. The research on localized temperature control using Cr nanowire (NW) arrays demonstrated the manipulation of thermal environments in NWs with a length of 2 μm, resulting in a temperature increase of the NWs to between 250 and 362 °C due to Joule heating [24]. ZnO-decorated Ag NWs also exhibited a temperature of ∼150 °C [25]. Furthermore, studies on Joule heating and mass transport in Au NWs have revealed intricate interactions between electrical currents and thermal dynamics, using finite element method simulations that show the influence of substrates on the temperature of the NWs [26]. However, it is noted that the temperature of metal nanoheaters tends to increase uniformly over the entire structure of the devices.

In addition, the semiconductor nanostructures were used as a building block for the development of nanoheaters [27], [28]. For example, Si NWs have been examined as nanoheaters, providing deeper insights into heat transport in integrated devices; the heat of ∼300 °C was distributed across the entire structure of NW devices at a micrometer scale [27]. Furthermore, the emergence of flexible and transparent electrothermal film heaters using graphene signifies a notable progress in the development of multifunctional nanoheaters, demonstrating improved Joule heating and localized light emission, essential for optoelectronic and thermal management applications [11], [19], [20], [28], [29]. However, these graphene-based nanoheaters also exhibited the heat transmission throughout the whole surface of graphene. Thus, the findings of nanoheaters using diverse materials and structures produced heat distributed across the entire device, which is inadequate for attaining the necessary performance of a point heater.

In this context, we develop a Si NW device featuring a porous Si segment with high resistance, serving as a nanoscale heat generator. The localized high-resistance segment enhances the device’s heat generation capacity while addressing thermal management issues at the sub-micrometer scale. We examine the thermal characteristics of the NW devices employing direct imaging techniques that visualize temperature profiles within the NW structures. These investigations demonstrate efficient heat generation at sub-micrometer scales, enabling the devices to function as point heaters.

## 2 Results

**Figure 1:**
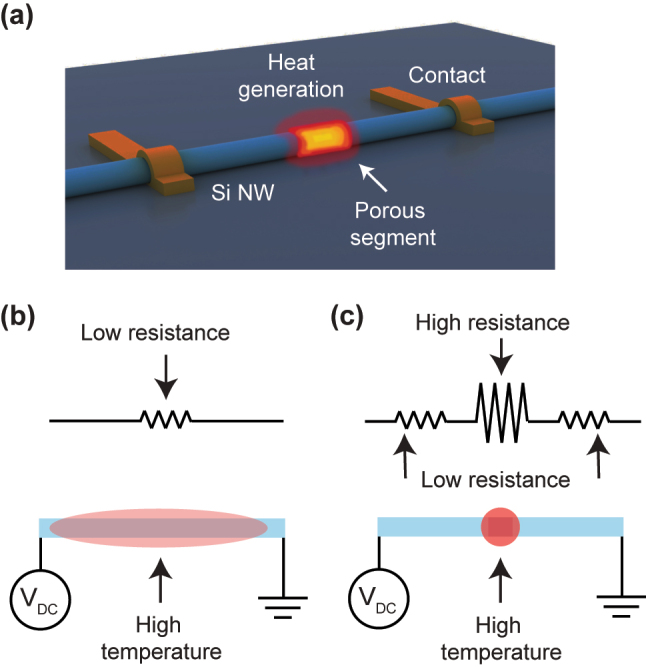
Nanoheater device. (a) Schematic of the nanoheater device utilizing a Si NW with a porous segment. Two electrical contacts are located on the solid segments of NW. Heat is locally generated on the porous segment. (b) Equivalent circuit of the single solid Si NW (top panel) and illustration of the NW (bottom panel). The NW is considered as a single low-resistance component, with the device producing heat uniformly across the entire NW. (c) Equivalent circuit of the single Si NW with the porous segment (top panel) and illustration of the NW heater using it (bottom panel). The high and low resistances in the equivalent circuit correspond to the porous and solid segments, respectively. Heat is mostly generated in the porous segment.

Figure 1a illustrates a schematic of a nanoheater device using a single Si NW including a porous segment. When an external voltage bias is applied to the electrical contacts on the NW, localized heat is generated within the porous segment. We compared the equivalent circuit of a solid Si NW and a Si NW with a porous segment to analyze local heat generation in the NW device. The homogeneous Si NW functions as a single resistor (top, Figure 1b). In this case, Joule heating produces uniform thermal distribution across the entire NW structure when applied by an external bias voltage (bottom, Figure 1b). On the other hand, a Si NW including a short porous segment positioned between two long solid segments is equivalent to a series connection of three resistors (top, Figure 1c). The solid and porous segments exhibit low and high resistance, respectively; the rough surface of the porous segment yields relatively higher resistance than the solid segment [30], [31]. Based on this model, we demonstrate nanoscale heat generation using a single Si NW with a porous segment.

**Figure 2:**
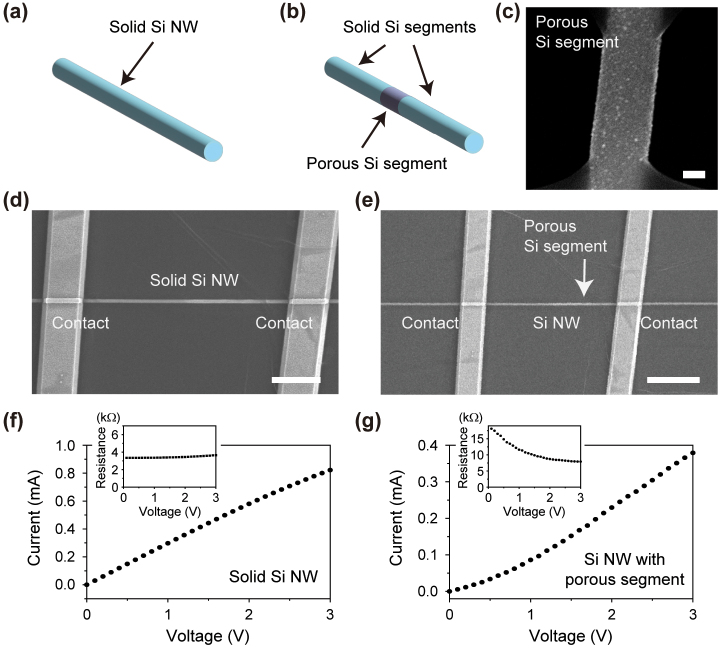
Si NWs without and with the porous segment. (a) Schematic of the solid single Si NW. (b) Schematic of the single Si NW with the porous segment between two solid segments. (c) SEM image of a porous segment of the Si NW. The scale bar is 100 nm. (d) SEM image of the solid Si NW device. The surface was passivated using hBN. The scale bar is 3 μm. (e) SEM image of the Si NW device with the porous segment. The surface was passivated using hBN. The position of the porous segment is indicated by a white arrow. The scale bar is 5 μm. (f–g) Measured *I-V* curves of the solid Si NW (f) and the Si NW with the porous segment (g). The insets show the resistance derived from the *I-V* curves as a function of voltage.

Figure 2a presents a schematic illustration of the solid Si NW, which features a uniform solid Si structure throughout its longitudinal axis. The NWs were dispersed on the Si_3_N_4_/SiO_2_/Si substrate and electrical contacts were fabricated on both ends of NWs. The scanning electron microscope (SEM) image of the solid Si NW device shows two electrical contacts on the NW, with a diameter of 250 nm and a channel length of 12 μm (Figure 2d). On the other hand, a Si NW with a porous segment, depicted in Figure 2b, was fabricated using a two-step metal assisted chemical etching (MaCE) (see Section 3) [32]. In the first step, the targeted regions of PMMA-coated Si NWs were opened using electron-beam (e-beam) lithography and subsequently immersed in a mixed solution of HF, AgNO_3_, and H_2_O to facilitate the formation of Ag nanoparticles (NPs) on the exposed NW surface. In the second step, a porous surface was formed by immersion in a mixed solution of HF, H_2_O_2_, and H_2_O with Ag NPs serving as an etching catalyst. The Ag NPs were removed using HNO_3_, remaining the porous segment in the NW. The porosity of the porous segment was optimized to generate an effective electrical current by controlling etching time (see Section 3). Figure 2c shows the SEM image of the porous segment, presenting the area of the NW that was exposed during the etching process, while the other surface was protected by an e-beam resist. The rough surface of the porous segment in NW was clearly observable after the second step of MaCE, with the porous segment spanning approximately 800 nm in length. The nanoheater was fabricated using these porous-segment embedded NWs by forming two electrical contacts. Figure 2e shows the SEM image of a porous-segment embedded NW device, featuring a diameter of 250 nm and a channel length of 12 μm. The porous Si segment, indicated by the white arrow in Figure 2e, was located between two electrical contacts.

To investigate the resistivity of the solid and porous segments in the Si NW, we measured their current-voltage (*I-V*) curves and converted them into resistance versus voltage plots (Figure 2f and g). Solid NWs exhibited linear characteristics, indicating that they operated as linear resistors (Figure 2f). The resistivity of the solid segment in the NW was determined to be 0.024 Ω cm, based on the diameter of 250 nm and the length of 12 μm of the solid Si NW. In contrast, the nonlinear *I*-*V* characteristics observed in the porous segment-embedded NW indicated a reduction in resistance within the 0–2 V region, maintaining nearly constant above 2 V (Figure 2g). The resistivity of the porous segment was estimated to be 0.442 Ω cm, taking into account the length fraction of the porous segment in the NW (see Section 3). In a series circuit with a constant electrical current, increased heat generation occurs in a higher resistance segment, as power is directly proportional to resistance. Consequently, localized heat can be primarily produced in the porous segment with a 19-fold increase in resistivity.

We note that the nonlinear *I-V* characteristic of the porous segment-embedded NW originates from its porous structure [30]. The porous segment is composed of microscopic networks of Si nanocrystal grains. At low voltages, the electron hopping process in the nanograin networks dominates the electrical current flow, leading to relatively high resistivity. On the other hand, at higher voltages, the space-charge limited current is formed by percolation paths in the nanograin networks, showing relatively low resistivity.

**Figure 3:**
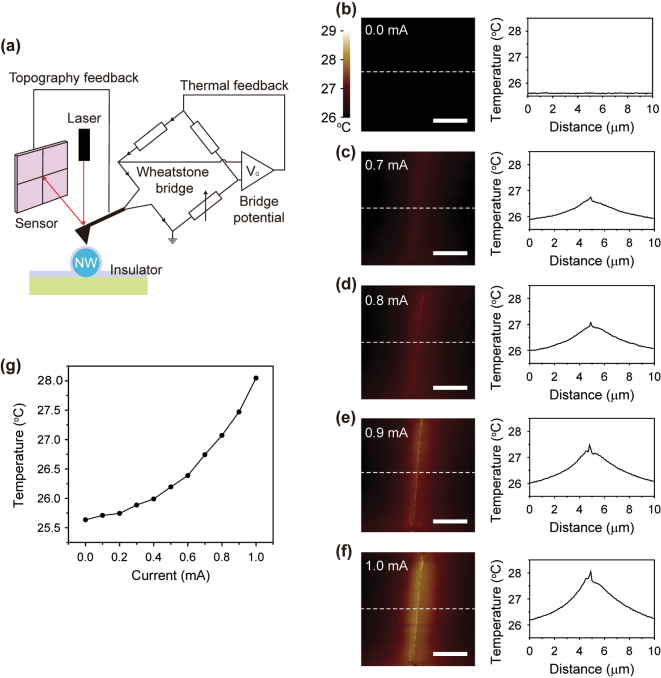
Measurement of heat generation in the solid Si NW. (a) Schematic illustration of SThM. The illuminating laser is reflected by the AFM tip and detected by the sensor to map the topography of the NW. The variable resistance in the Wheatstone bridge, connected to the tip, is adjusted under the bridge potential, and the temperature change is estimated by the potential change. (b–f) SThM images (left panels) and temperature profiles (right panels) of the solid Si NW device. The current was applied to the device at 0.0 mA (b), 0.7 mA (c), 0.8 mA (d), 0.9 mA (e), and 1.0 mA (f). The colored scale bar indicates the measured temperature. The scale bars are 3 μm in each left panel. The temperature profile was obtained from the white dashed lines in each SThM image. (g) Temperature curve as a function of the current. The temperature was obtained at the peak of the measured temperature profiles.

We conducted scanning thermal microscopy (SThM) to examine heat generation and heat dispersion around the prepared NW devices while electrical current was applied (Figure 3a). The SThM, utilizing the contact mode of AFM, measures an output potential change of a Wheatstone bridge connected to an AFM tip, with temperature changes inferred from variable resistance changes. Thus, it is essential to passivate the surfaces of devices with insulators. We transferred thin hexagonal boron nitride (hBN) layers onto the fabricated devices by the wet transfer method. hBN has the advantages of being a good insulator and exhibiting a high flexibility, making it one of the ideal materials for passivating the surfaces with considerable height undulation in devices by a simple manner.

First, we mapped the temperature distribution of the solid NW device while controlling the electrical current from 0.0 to 1.0 mA. The spatial resolution is 200 nm. At 0.0 mA, the uniform room temperature was recorded over the entire area (left, Figure 3b). The temperature profile across the NW device also indicates a consistent 25.6 °C (right, Figure 3b). A slight temperature increase was observed from the NW device within the range of 0.1–0.6 mA. Starting at 0.7 mA, a bright vertical bar shape was seen in the temperature map, corresponding to the NW configuration (left, Figure 3c). The temperature profile indicated 26.8 °C in the NW, exceeding the 0.0 mA case by 1.2 °C (right, Figure 3c). The temperature decreased gradually from the top of the NW to the outside, and eventually reaching 25.9 °C at a distance of approximately 5 μm from the NW. At 0.8 mA, the bright bar shape exhibited increased contrast, with a maximum temperature measured at 27.1 °C (Figure 3d). At 0.9 and 1.0 mA, temperature maxima were observed in the temperature map when the NW was present (left, Figure 3e and f). The highest temperatures recorded were 27.5 °C at 0.9 mA and 28.0 °C at 1.0 mA, respectively (right, Figure 3e and f).

A uniform temperature distribution was observed along the NW axis. On the other hand, the temperature profile across the NW cross-section reveals pronounced high temperature peaks that correlate to the NW structure, which are around 0.2–0.3 °C higher than the surrounding region. Consequently, the extensive high temperature distribution is dominated by the longitudinal axis and diameter of the NW, which is spatially defined at the microscale. Furthermore, to examine the temperature variations in relation to the applied current, we plotted the peak temperature as a function of the current. As the current increased from 0.0 to 1.0 mA, the temperature increased from 25.6 to 28.0 °C in a quadratic parabola, corroborating that the heat was generated via Joule heating (Figure 3g).

**Figure 4:**
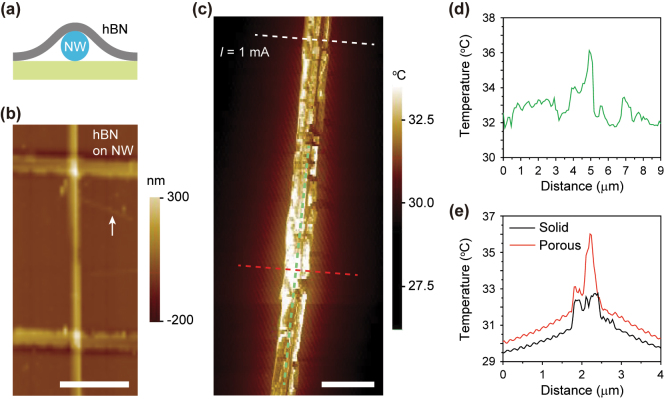
Heat generation in the hBN-passivated NW. (a) Cross-sectional illustration of the hBN-covered Si NW on the substrate. (b) AFM image of the Si NW device. The surface was passivated using hBN. The white arrow denotes the wrinkle of the hBN layer. The colored scale bar indicates the measured height. The scale bar is 4 μm. (c) SThM image of the Si NW device with the porous segment in b. A current of 1.0 mA was applied to the NW device. The colored scale bar indicates the measured temperature. The scale bar is 2 μm. (d) Temperature profile along the longitudinal axis of the NW device (green dashed line in c). The temperature maximum was recorded at ∼5 μm, which is the location of the porous segment. (e) Temperature profiles along the cross-sectional axes of the NW device in the solid (black) and porous (red) segments. The black and red curves were measured along the white and red dashed lines in c, respectively. The temperature maxima were observed at ∼2 μm, which is the location of the NW. The higher temperature peak was recorded at the porous segment.

We utilized the porous segment-embedded Si NW to achieve geometric confinement of heat generation along the longitudinal and cross-sectional axes of the NW. We selected hBN for surface passivation; during the wet transfer of hBN onto the NW, an air gap may develop between the substrate and the suspended hBN, as illustrated in Figure 4a. Figure 4b presents the AFM topographical image of the NW, revealing a device structure with well-adhered hBN. The surface of the hBN-coated substrate was smooth, although a wrinkle caused by the transfer process was observed near the NW, as indicated by the white arrow in Figure 4b.

Next, SThM measurements were conducted on the NW device with an electrical current of 1.0 mA. Figure 4c shows the heat mapping image of the NW device. The porous segment has significantly brighter contrast than the surrounding region. To quantitatively examine the heat dispersion, temperature profiles were plotted along the longitudinal axis of the NW (Figure 4d) and across two cross-sectional axes of the solid and porous segments (Figure 4e). The temperature profile along the NW axis (green dashed line, Figure 4c) reveals a distinct peak of 36.1 °C at the porous segment, whereas the temperature in the solid segment remains rather constant ranging from 32.0 to 33.0 °C (Figure 4d). The cross-sectional temperature profiles contrasted the solid (white dashed line, Figure 4c) and the porous segments (red dashed line, Figure 4c). A temperature peak of 36.0 °C was recorded in the porous segment, whereas the solid segment has a peak of 32.7 °C (Figure 4e). Taken together, the porous segment of the NW generated approximately 3.3 °C more heat than the solid segment. Furthermore, according to the measured temperature profiles, the heat was localized not only along the cross-sectional axis but also along the longitudinal axis of the NW at a distance of less than 1 μm.

**Figure 5:**
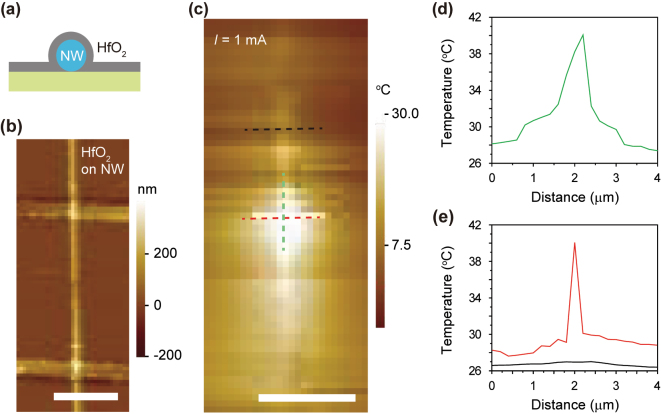
Heat generation in the HfO_2_-passivated NW. (a) Cross-sectional illustration of the HfO_2_-passivated Si NW on the substrate. The HfO_2_ layer is conformally coated on the NW. (b) AFM image of the Si NW device with the porous segment. The surface was passivated using HfO_2_. The colored scale bar denotes the height of surface. The scale bar is 5 μm. (c) SThM image of the Si NW device in b. A current of 1.0 mA was applied to the NW device. The bright contrast was concentrated in the porous segment. The colored scale bar indicates the measured temperature. The scale bar is 5 μm. (d) Temperature profile along the axis of the NW device indicated by the green dashed line in c. The temperature maximum was observed at ∼2 μm, which is the location of the porous segment. (e) Temperature profiles along the cross-sectional axes of the NW device at the solid (black) and porous (red) segments. The black and red curves were measured along the black and red dashed lines in c, respectively. The temperature maxima were observed at ∼2 μm. The higher temperature peak was recorded at the porous segment.

To minimize the lack of full conformality of the hBN layer and investigate the influence of a more conformal passivation layer on the NW device, we repeated the experiment by applying an alternative insulating material [33]. Instead of using hBN as a passivation layer, the NW heater was fabricated with a passivation layer of 30 nm thick HfO_2_ which was deposited using atomic layer deposition (ALD). We note that the length and diameter of the porous segment were 800 nm and 250 nm, respectively, consistent with those in Figure 4. The ALD process ensures conformal passivation across an extended area, effectively eliminating any air gaps between the HfO_2_ layer and the NW on the substrate, as depicted in Figure 5a.

Figure 5b shows the AFM image of the device. The conformal surface was observed without any wrinkles. The topological profile indicates minimal surface roughness of a few nanometers. We assessed the SThM of the device coated with HfO_2_ at an applied current of 1.0 mA, demonstrating the concentration of heat dispersion in the porous segment (Figure 5c). To quantitatively analyze the temperature, the temperature profiles were displayed along the longitudinal and cross-sectional axes (Figure 5d and e). The heat was localized within 1 μm along the longitudinal axis of the NW, exceeding the surrounding substrate temperature by more than 12.0 °C (Figure 5d). A rapid temperature drop occurs from 40.0 °C to ∼28.0 °C within 2 μm distance of the porous segment of the device. Along the cross-sectional axis of the NW, the temperature reaches 40.0 °C in the NW (red line, Figure 5e), exceeding the surrounding substrate temperature of ∼30.0 °C by more than 10.0 °C within a distance of only 400 nm. The temperature profile approximately 2 μm from the porous segment exhibits uniformity, ranging from 26.0 to 27.0 °C (black line, Figure 5e). Consequently, the HfO_2_-passivated device demonstrated superior heat localization compared to that coated with hBN. This enhancement can be attributed to the uniform adherence of the passivation layer to the NW device.

In conclusion, we demonstrated efficient heat generation within a confined sub-micrometer area in the porous segment-embedded Si NW device, functioning as a point heater. Heat generation primarily occurred along the longitudinal axis of the homogeneous solid Si NW, while its dispersion was effectively limited to the cross-sectional axis. To enhance heat confinement along the longitudinal axis, we incorporated a high-resistance porous segment in the middle of the Si NW. Given that the porous section exhibits 19 times more resistivity than the solid segment, this design promoted predominant Joule heating within the porous segment of the NW. In the experiment, SThM measurements provided clear evidence of localized heat generation by directly visualization of heat distribution. The hBN-passivated Si NW device with the porous segment produced a temperature increase of 3.3 °C at a sub-micrometer scale. Furthermore, the HfO_2_-passivated NW device demonstrated a temperature increase exceeding 10 °C within a 0.4 μm × 1 μm area. We believe that the porous segment-embedded NW point heater can show considerable potential for future applications, particularly in biomedical engineering such as localized cell therapy and drug delivery systems.

## 3 Methods

**Fabrication of nanoheaters.** Si nanowires (NWs) with diameters of 250 nm were synthesized at 480 °C for 60 min via a chemical vapor deposition process under a total pressure of 40 Torr (174 sccm H_2_, 2 sccm SiH_4_, 20 sccm PH_3_, and 4 sccm HCL). The NWs, characterized by a high n-type doping level, were dispersed onto a Si_3_N_4_/SiO_2_/Si substrate. The porous segments in the Si NWs were formed by two-step metal-assisted chemical etching process. The region designated as a porous segment was defined by electron-beam (e-beam) lithography. C6 PMMA e-beam resistor was used. In the first etching step, the prepared samples were immersed into a solution of HF, AgNO_3_, and H_2_O [v:v:v = 1:0.015:100] for 12 s at room temperature to form Ag nanoparticles (NPs) on the surface. In the second etching step, the Ag NPs-decorated NWs were immersed into a solution of HF, H_2_O_2_, and H_2_O [v:v:v = 1:1:20] for 10 s at room temperature. This was the optimal etching time, considering the effective electrical current in the NW device. Longer etching times resulted in exceedingly low currents, measuring only a few pA for a 20-s etching time [32]. The Ag NPs were subsequently removed using HNO_3_ for 10 s. After removing the PMMA by acetone for 10 m, the porous segments were formed onto the Si NWs.

To fabricate electrical contacts at the ends of the NWs, the contact regions were defined using e-beam lithography, and 300 nm of gold was deposited by thermal evaporation. hBN and HfO_2_ were used for insulation between the NW and the SThM tip. hBN was applied to the NWs by a PMMA-assisted transfer method. HfO_2_ with a thickness of 30 nm was coated on the NWs using atomic layer deposition (ALD) process.

**Measurement of resistivity.** The *I-V* measurements were conducted using a source measure unit (SMU; 2450 SourceMeter, Keithley) and a probe station. The current was recorded while the voltage was varied from 0 to 3 V. We obtained the resistivities of the solid and porous segments using Ohm’s law, *R* = *V*/*I*, and the resistivity equation, *ρ* = *RA*/*l*, where the *R*, *V*, *I*, *ρ*, *A*, and *l* are resistance, voltage, current, resistivity, area, and length. The resistivity of the solid segment (*ρ*_solid_) was determined to be 0.024 Ω cm, from the *I*-*V* curve (Figure 2f) along with the structural dimensions of 250 nm in diameter and 12 μm in length. For the porous segment, we considered the length fraction of the porous segment in the NW using the equation, *V*/*I* = *ρ*_solid_ *l*/*A* (11.2/12) + *ρ*_porous_ *l*/*A* (0.8/12), where the lengths of the entire NW, the solid segment, and the porous segment were 12 μm, 11.2 μm, and 800 nm, respectively. Then, from the *I*-*V* curve (Figure 2g), we determined the resistivity of the porous segment (*ρ*_porous_) to be 0.442 Ω cm at 3 V.

**SThM measurements.** We performed the scanning thermal microscopy (SThM) measurements using Park NX-10. The temperature calibration of the SThM tip was carried out by increasing its temperature from room temperature to 60 °C and contacting the tip with a thermoplastic polyurethane film that melts within the range of 50 and 60 °C. The contact mode was used to measure the temperature of the nanoheater. The sample surface was scanned at intervals of 200 nm. The SThM quantifies an output voltage variation from a Wheatstone bridge connected to an AFM tip, with temperature change inferred from resistance variation. The electrical source/measurement unit (Keithley 2450) was used to supply current to the nanoheater.
